# Supplementary material for: From sequence to enzyme mechanism using multi-label machine learning
Source: BMC Bioinformatics. 2014 May 19;15:150. doi: 10.1186/1471-2105-15-150 (PMC4229970; doi:10.1186/1471-2105-15-150)
Supplement: Additional file 2 — Java code of ml2db. Additional file ml2db_code.tar.gz contains the Java source code to run the multi-label machine learning experiments and save the results to database. The code’s Javadoc is included. [file 1471-2105-15-150-S2.zip › additional file 2/ml2db/utils/doc/edu/cornell/lassp/houle/RngPack/Ranmar.html]

Ranmar


JavaScript is disabled on your browser.


- Overview
- Package
- Class
- Use
- Tree
- Deprecated
- Index
- Help

- Prev Class
- Next Class

- Frames
- No Frames

- All Classes

- Summary:
- Nested |
- Field |
- Constr |
- Method

- Detail:
- Field |
- Constr |
- Method


edu.cornell.lassp.houle.RngPack

## Class Ranmar

- java.lang.Object
- - cern.colt.PersistentObject
  - - edu.cornell.lassp.houle.RngPack.RandomElement
    - - edu.cornell.lassp.houle.RngPack.RandomSeedable
      - - edu.cornell.lassp.houle.RngPack.Ranmar

- All Implemented Interfaces:
  :   java.io.Serializable, java.lang.Cloneable

  ---

    

  ```
  public class Ranmar
  extends RandomSeedable
  ```

  RANMAR is a lagged Fibonacci generator proposed by Marsaglia and
  Zaman and is a good research grade generator. This version of RANMAR
  is based on the paper by James, which is a good reference for the properties
  of RANMAR and several other generators.
    
  **REFERENCES:**   
  F. James, Comp. Phys. Comm. **60** (1990) p 329-344   
  and was originally described in   
  G. Marsaglia, A. Zaman and W.-W Tsang, Stat. Prob. Lett
  **9** (1990) p 35.  More
  info .

  Source code  is available.

  Version:
  :   1.0

  Author:
  :   Paul Houle  (E-mail: houle@msc.cornell.edu)

  See Also:
  :   Serialized Form

- - ### Field Summary

    Fields

    | Modifier and Type | Field and Description |
    | --- | --- |
    | `static int` | `BIG_PRIME` The 46,009,220nd prime number, he largest prime less than 9\*10^8^. |
    | `static int` | `DEFSEED` Default seed. |
  - ### Constructor Summary

    Constructors

    Constructor and Description

    `Ranmar()`

    Initialize Ranmar with a default seed taken from Marsaglia and Zaman's
    paper.

    `Ranmar(java.util.Date d)`

    Seed RANMAR from the clock.

    `Ranmar(int ijkl)`

    Initialize Ranmar with a specified integer seed

    `Ranmar(long ijkl)`

    Initialize Ranmar with a specified long seed
  - ### Method Summary

    Methods

    | Modifier and Type | Method and Description |
    | --- | --- |
    | `java.lang.Object` | `clone()` Returns a copy of the receiver; the copy will produce identical sequences. |
    | `double` | `raw()` Returns a uniformly distributed pseudo-random double in the range (0,1). |
    | `void` | `raw(double[] d, int n)` A version of the generator for filling arrays, inlined for speed |

    - ### Methods inherited from class edu.cornell.lassp.houle.RngPack.RandomSeedable

      `ClockSeed, ClockSeed`
    - ### Methods inherited from class edu.cornell.lassp.houle.RngPack.RandomElement

      `choose, choose, gaussian, gaussian, powlaw, raw, uniform`
    - ### Methods inherited from class java.lang.Object

      `equals, getClass, hashCode, notify, notifyAll, toString, wait, wait, wait`

- - ### Field Detail


    - #### BIG\_PRIME

      ```
      public static int BIG_PRIME
      ```

      The 46,009,220nd prime number, he largest prime less than
      9\*10^8^. Used as a modulus because this version of
      RANMAR needs a seed between 0 and 9\*10^8^ and
      `BIG_PRIME` isn't commensurate with any regular period.
      `BIG_PRIME= 899999963`


    - #### DEFSEED

      ```
      public static int DEFSEED
      ```

      Default seed. `DEFSEED=54217137`
  - ### Constructor Detail


    - #### Ranmar

      ```
      public Ranmar()
      ```

      Initialize Ranmar with a default seed taken from Marsaglia and Zaman's
      paper. Equivalent to `Ranmar(54217137).`


    - #### Ranmar

      ```
      public Ranmar(java.util.Date d)
      ```

      Seed RANMAR from the clock.

      ```
       RandomElement e = new Ranmar(new Date());
      ```

      Parameters:
      :   `d` - a Date object to seed Ranmar with, typically
          `new Date()`


    - #### Ranmar

      ```
      public Ranmar(int ijkl)
      ```

      Initialize Ranmar with a specified integer seed

      Parameters:
      :   `ijkl` - seed integer; Ranmar(int ijkl) takes uses
          ijkl modulus BIG\_PRIME as a seed for
          RANMAR.


    - #### Ranmar

      ```
      public Ranmar(long ijkl)
      ```

      Initialize Ranmar with a specified long seed

      Parameters:
      :   `ijkl` - seed long; Ranmar(long ijkl) takes uses ijkl
          modulus BIG\_PRIME as a seed for RANMAR.
  - ### Method Detail


    - #### clone

      ```
      public java.lang.Object clone()
      ```

      Returns a copy of the receiver; the copy will produce identical
      sequences. After this call has returned, the copy and the receiver have
      equal but separate state.

      **Overrides:**
      :   `clone` in class `PersistentObject`

      Returns:
      :   a copy of the receiver.


    - #### raw

      ```
      public final double raw()
      ```

      Returns a uniformly distributed pseudo-random double in the range (0,1).

      **Specified by:**
      :   `raw` in class `RandomElement`

      Returns:
      :   a pseudo random number in the range (0,1).

      See Also:
      :   `RandomJava`


    - #### raw

      ```
      public final void raw(double[] d,
             int n)
      ```

      A version of the generator for filling arrays, inlined for speed

      **Overrides:**
      :   `raw` in class `RandomElement`

      Parameters:
      :   `d` - an array of doubles to be filled
      :   `n` - size of the array


- Overview
- Package
- Class
- Use
- Tree
- Deprecated
- Index
- Help

- Prev Class
- Next Class

- Frames
- No Frames

- All Classes

- Summary:
- Nested |
- Field |
- Constr |
- Method

- Detail:
- Field |
- Constr |
- Method
